# Histological Alterations in Hashimoto’s Disease: A Case-Series Ultrastructural Study

**DOI:** 10.3390/medicines10090051

**Published:** 2023-09-02

**Authors:** Eleni Avramidou, Antonios Gkantaras, Iasonas Dermitzakis, Konstantinos Sapalidis, Maria Eleni Manthou, Paschalis Theotokis

**Affiliations:** 1 Department of Histology-Embryology, School of Medicine, Aristotle University of Thessaloniki, 54124 Thessaloniki, Greece; avramidoue@auth.gr (E.A.); iasonasd@auth.gr (I.D.); mmanthou@auth.gr (M.E.M.); 2 Pediatric Immunology and Rheumatology Referral Centre, First Department of Pediatrics, Ippokration General Hospital, Aristotle University of Thessaloniki, 54124 Thessaloniki, Greece; antonisgk95@gmail.com; 3 3rd Surgical Department, “AHEPA” University Hospital, School of Medicine, Aristotle University of Thessaloniki, 54124 Thessaloniki, Greece; sapalidiskonstantinos@gmail.com

**Keywords:** Hashimoto’s thyroiditis, electron microscopy, ultrastructure, blood vessels, angiogenesis

## Abstract

Background: Hashimoto’s thyroiditis (HT) is an autoimmune disease exhibiting stromal fibrosis and follicular cell destruction due to lymphoplasmacytic infiltration. Besides deprecated analyses, histopathological approaches have not employed the use of electron microscopy adequately toward delineating subcellular-level interactions. Methods: Biopsies for ultrastructural investigations were obtained from the thyroids of five patients with HT after a thyroidectomy. Transmission electron microscopy (TEM) was utilized to study representative tissue specimens. Results: Examination indicated interstitial extravasated blood cells and a plethora of plasma cells, based on their subcellular identity landmarks. These antibody-secreting cells were profoundly spotted near follicular cells, fibroblasts, and cell debris entrenched in collagenous areas. Pathological changes persistently affected subcellular components of the thyrocytes, including the nucleus, endoplasmic reticulum (ER), Golgi apparatus, mitochondria, lysosomes, and other intracellular vesicles. Interestingly, significant endothelial destruction was observed, specifically in the larger blood vessels, while the smaller vessels appeared comparatively unaffected. Conclusions: Our TEM findings highlight the immune-related alterations occurring within the thyroid stroma. The impaired vasculature component and remodeling have not been described ultrastructurally before; thus, further exploration is needed with regards to angiogenesis in HT in order to achieve successful prognostic, diagnostic, and treatment-monitoring strategies.

## 1. Introduction

Hashimoto’s thyroiditis (HT), or chronic lymphocytic thyroiditis, is a chronic autoimmune, inflammatory disease of the thyroid gland, also known as “struma lymphomatosa” [1]. Its main histologic feature is thyroid infiltration by lymphocytes and plasma cells, as well as the destruction of follicles [2]. HT constitutes the most frequent cause of spontaneous hypothyroidism in iodine-sufficient areas and concerns a large part of the world’s population [3]. It is most commonly found in women, especially those of premenopausal age [4]. A continuous linear increase in the annual frequency of HT for almost twenty years was demonstrated in 2010 [5]. As pointed out in the Whickham survey [4], 8% of women and 3% of men in the general population had subclinical hypothyroidism, confirming previously reported results from similar studies [4]. In populations with severe iodine deficiency, the prevalence of hypothyroidism is even higher [3].

In the early stages of the disease, namely subclinical hypothyroidism, antibodies against thyroperoxidase (TPO) and higher thyroid-stimulating hormone (TSH) levels are detected. When clinical hypothyroidism develops, T3 and T4 thyroid hormone levels begin to drop [6]. An HT patient is advised to check thyroid hormones and TPO antibodies at least once or twice per year to adjust the administered dose of thyroid hormones [7]. Hypothyroidism caused by HT may cause many health problems, such as fatigue, cold intolerance, weight gain, and depression. If left untreated, HT may lead to goiter, heart conditions, and myxedema [8]. Hashimoto has been associated with a higher risk of type 1 diabetes, malabsorption disorders, infertility issues, mostly in women, and a higher prevalence of thyroid cancer [9].

One of the primary features of the HT ultrastructure is the abundant inflammatory infiltration within the stroma, both in follicular and parafollicular areas [10,11,12,13]. The cytotoxic reaction against thyrocytes is mediated by autoantigen presentation, and the high numbers of CD8^+^ cells and plasma cells lead to epithelial cell destruction and fibrosis [14]. Although HT is well studied, the exact pathophysiological processes are not yet fully elucidated, similar to other autoimmune diseases [14]. A limited number of studies focus on describing ultrastructural changes detected with transmission electron microscopes (TEMs), with the most recent being held in 2011 [10,11,12,13,14,15,16,17,18,19,20,21,22,23,24,25]. Therefore, updated information regarding the ultrastructural pathology of this condition is essential for contributing to delineating HT’s precise pathophysiology and accurately determining the level of organ functionality [10,11,12,13,14,15,16,17,18,19,20,21,22,23,24,25,26].

The aim of our study was to present updated data on thyroid ultrastructure in Hashimoto’s disease. In the current report, we focused on and gained new insight into vessels’ morphological traits, which, to our knowledge, have not been previously described in detail. Finally, we discuss the potential role of these traits in the pathogenesis of HT.

## 2. Materials and Methods

### 2.1. Subjects

The study involved five female patients, aged 45–50 years, affected by Hashimoto’s thyroiditis. They all exhibited hypothyroidism as well as an elevated TSH and were treated with L-thyroxine for more than two years. Before surgery, patients had signed informed consent forms provided by the surgical department. They were operated on because of the intensive growth of the goiter, whereas all of them were in a euthyroidism state at the time of excision. The thyroid specimens were immediately sent for histological investigation, with the surgical pathology report confirming the presence of HT.

### 2.2. Electron Microscopy

Samples were collected immediately following a thyroidectomy to be used for ultrastructural investigations. Small segments of tissue, measuring 0.5 mm^3^, were taken and promptly prepared for transmission electron microscopy. The electron microscopy analysis was conducted at the Laboratory of Histology and Embryology at the School of Medicine of Aristotle University of Thessaloniki.

All specimens were fixed in a pH 7.4 buffered solution containing 4% glutaraldehyde for a duration of 90 min. They were subsequently postfixed in a 2% OsO_4_ solution in the same buffer for 1 h at room temperature. The specimens were then dehydrated by progressively increasing the concentration of ethanol until a saturation of 100%. Finally, the specimens were embedded in Epon 812 and polymerized overnight at 60 °C. The Epon blocks were cut using an ultramicrotome (Leica EM UC6).

### 2.3. Quantitative Morphometric Data and Statistics

The number of fibroblasts amongst thyrocytes (or within follicles) was counted and scaled to cells per square millimeter (3 areas per case; spaced at least 50 μm apart). Likewise, a spatial morphometric analysis was carried out, and the percentages of these active cells and the less active ones (fibrocytes) were evaluated with regard to their proximity to a vessel (proximal or distal). The analysis of the data was performed using the GraphPad Prism 8.0 software. The assessment of normal distribution was performed utilizing the Shapiro–Wilk and Kolmogorov–Smirnov tests. Parametric data underwent analysis via an unpaired *t*-test. Mean ± standard error was employed to express values for all scaled data. The level of significance was set at *p* < 0.05.

## 3. Results

### 3.1. Immune Response in the Thyroid Gland Interstitium

**Figure 1:**
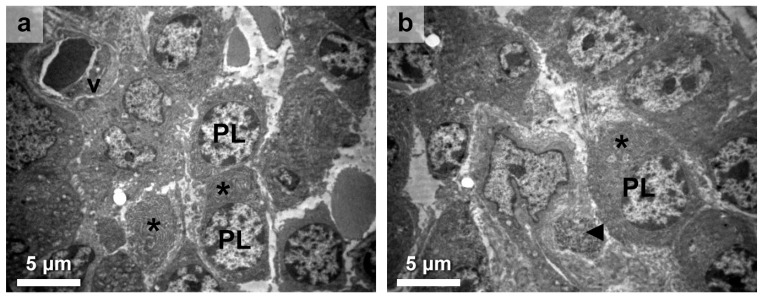
Immune cells identified with TEM in the HT thyroid gland stroma. (**a**). Lymphocytes and plasma cells infiltrate the interstitium surrounding the affected thyroid follicles, where plasma cells (PLs) actively attack the follicular cells. The prominent endoplasmic reticulum and Golgi apparatus (asterisk) indicate protein synthesis and secretion. A vessel (v) is present nearby among thyrocytes. (**b**). Additionally, a macrophage with intracellular debris (arrow) is observed, suggesting its involvement in phagocytic processes. Plasma cells (PLs) were also documented based on ER appearance (asterisk). Scales are denoted in each micrograph.

Our investigation into the immune aspects of HT revealed events regarding the immune response in the interstitium, specifically highlighting the presence of lymphocytes and, notably, plasma cells actively attacking the thyroid follicles (Figure 1a). Moreover, a detailed analysis of plasma cell ultrastructure revealed prominent and enlarged endoplasmic reticulum (ER) and Golgi apparatus (GA), with wider cisternae (Figure 1b; asterisk), suggesting significant alterations in protein synthesis and secretion within these cells. Additionally, we observed the presence of a macrophage containing intracellular debris (Figure 1b; arrow), indicating its involvement in phagocytic processes within the interstitium. These findings provide insight into the potential enhanced production and secretion of antibodies by plasma cells, as well as the role of macrophages in clearing cellular debris in the immune response associated with HT.

### 3.2. Thyroid Follicular Cell Alterations

**Figure 2:**
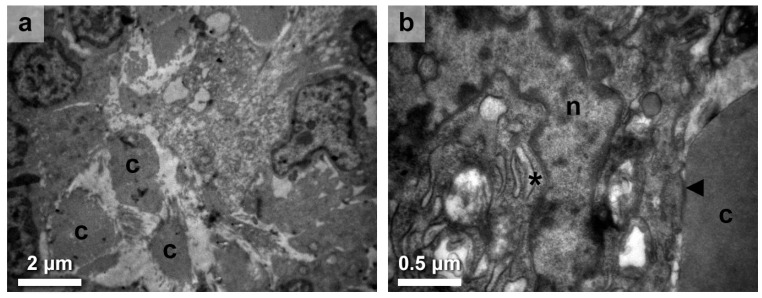
Thyroid epithelium and follicular impairment. (**a**). Thyroid follicular cells displayed changes characterized evidently by colloid decomposition appearing as droplets (c) within a follicle. (**b**). A higher magnification image provides a closer look at the cellular changes, revealing the irregular nucleus (n), enlarged rough endoplasmic reticulum, distended with wider cisternae (asterisk), and other intracellular organelles with abnormal membranes. Furthermore, the degenerated epithelium demonstrates an evident loss of microvilli (arrow) toward the apical surface, which is in direct contact with the colloid. Scales are denoted in each micrograph.

The majority of HT specimens yielded significant findings regarding the pathology and cellular alterations within the thyroid follicles. The observed follicular destruction was marked by pronounced colloid decomposition (Figure 2a), indicative of compromised follicular integrity and functionality. The thyroid follicles were generally smaller than average, and colloid was limited, appearing as droplets between thyrocytes. Apart from the invasion of follicles by plasma cells and lymphocytes, fibroblasts were also observed among thyrocytes in the interfollicular areas.

Fibroblasts were quite often, individually or in groups, situated very close to small vessels (178 ± 9.8 cells/mm^2^). Quantitative analysis of the active fibroblasts showed a significantly increased percentage (31.8 ± 2.2%) of them being in close proximity to the vessels, as opposed to being distal to them (6.8 ± 2.8%; unpaired *t*-test; *p* < 0.001). This location-based finding was also verified when fibrocytes (the less active ones based on the richness of cytoplasm) were found to be significantly less proximal than the ones distal to the vessels (29.8 ± 3.2% versus 18.6 ± 2.4%; unpaired *t*-test; *p* < 0.01). This is an especially remarkable observation, considering that the area further away from the vessel is often free of fibroblasts, suggesting an affinity of fibroblasts toward vessels.

Through the analysis of thyrocytes at the ultrastructural level, notable abnormalities were identified in cellular structures and organelles. The nuclei were of highly irregular shape (Figure 2b). Mitochondria appeared larger, more oval, or rounder than expected, with a denser matrix accompanied by lengthened and dilated cristae. The rough ER was prominent, enlarged, and distended with wider cisternae (Figure 2b; asterisk). The cells’ cytoplasmic membrane had an abnormal shape invading within the underlying basal lamina, which was found to be thickened. Lastly, the degenerated epithelium had an obvious loss of microvilli toward the apical surface, the one in close contact with the colloid (Figure 2b; arrow). The persistent alterations in these subcellular structures highlight the widespread impact of HT on key cellular processes involved in thyroid homeostasis.

### 3.3. Eclectic Endothelial Perturbations

**Figure 3:**
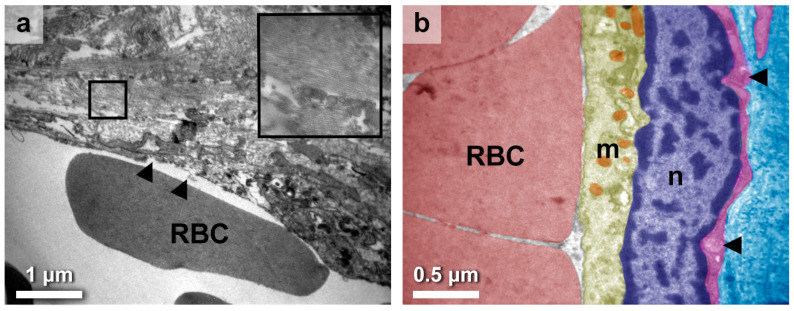
Endothelial cell alterations at the ultrastructural level. (**a**). Large vessels exhibited extensive damage, showcasing ruptures on the endothelium (arrows) and displaced collagen fibers within the lumen. Additionally, fibroblasts and collagen fibers (inset) were observed in the vicinity of the damaged endothelium, contributing to fibrotic scarring. (**b**). Endothelial cells exhibited nuclei (n) with irregular outlines, whereas their mitochondria (m) appeared to be denser and smaller in size, implying a perturbed energy metabolism within their cell bodies. Additionally, the majority of endothelial cells displayed disrupted basal lamina, with noticeable alterations and a lack of continuity with the underlying reticular lamina (arrows). Transversely cut collagen fibers can be seen underneath as dark dots. Micrograph pseudocolors: red = red blood cell (RBC), yellow = endothelial cell cytoplasm, orange = mitochondria, purplish blue = endothelial cell nucleus, magenta = basal lamina, and cyan = thyroid intermediate connective tissue. Scales are denoted in each micrograph.

Inflammatory cells within the interstitium exhibited an additional adverse impact on the vascular system of the gland. More specifically, most of the large thyroid vessels appeared damaged, even areas lacking endothelium (Figure 3a), whereas the small vessels displayed fewer impairments. There could be small ruptures present on the endothelium or displaced collagen fibers within the lumen, indicating larger damage nearby. Fibroblasts, collagen fibers as bundles (Figure 3a; inset), and miscellaneous cell debris were also found around the damaged endothelium.

Endothelial cells appeared with particular altered characteristics, such as lobular nuclei with irregular outlines (Figure 3b). Mitochondria exhibited dark matrixes and small size, suggesting perturbations in the energy metabolism. In most cases, the endothelium showcased a basal lamina that was disrupted, exhibiting alterations, and was not continuous with the underlaid reticular one (Figure 3b; arrow). Importantly, these unique findings could only be discerned through ultrastructural examination, representing a novel contribution within the present study.

## 4. Discussion

The current ultrastructural study regarding HT revealed the presence of small vessels without significant damage and the existence of larger vessels with extended impairments in the thyroid parenchyma. To the best of our knowledge, this is the first study to describe the vascular alterations in HT. The presence of collagen fibers, fibrous bundles, and basement membrane material in close association with degenerated thyroid follicles indicates thyroid damage in HT [11,25]. It was suggested that the great amounts of collagen surrounding thyroid follicles disrupt the exchange between capillaries and thyrocytes, resulting in cell death [14]. Our findings support the idea that this exchange may be impeded due to structural alterations of vessels as well, not only by collagen deposition.

The most described microscopical abnormalities of HT are lymphocytic infiltration in the stroma and oxyphilic change of the follicular epithelium [10,11,12,13]. The immune cells identified in HT are mostly lymphocytes and plasma cells, whereas the presence of macrophages and giant cells is also reported [11,21]. In certain cases, careful examination of the ultrastructure reveals connections between lymphocytes and either plasmacytes or thyrocytes, resembling an immunological synapse and indicating their interactions [14,15,25]. Interestingly, our results corroborate the existence of stromal lymphocytes, plasma cells, which were found to exhibit increased capacity for antibody production based on the appearance of the cytosolic ER–GA, and sparse macrophages. The lymphoid tissue additionally exhibits large B-cell-rich follicles with prominent germinal centers, substantiating the employment of connective tissue wandering cells, such as plasma cells.

The existing limited literature on the ultrastructural pathology of HT describes various degrees of alterations of the thyroid follicular cells in autoimmune thyroiditis, possibly reflecting different timepoints of the sequential processes of immune-mediated thyroid cell destruction [10,27]. As the present study indicated, thyrocytes in HT, even in the same follicle, vary in shape and size, as well as in the content and morphology of intracellular organelles [12,15]. The predominant alterations are observed in mitochondria, which, in many cases, appeared to be swollen, elongated with indistinct cristae, and denser matrix, as well as in the endoplasmic reticulum, which tended to be enlarged with dilated cisternae [11,15,21]. Various efforts to classify and name the altered epithelial cells encountered in HT were made without succeeding in adopting common pathological terminology, except for the widely used term “typical oncocyte”, which describes altered thyrocytes with an abundance of enormous mitochondria [10,11,21].

The current ultrastructural study has yielded interesting insights with potential implications for clinical practice. The identification of altered thyroid follicles and their impact on capillary–thyrocyte exchange underscores structural alterations that extend beyond conventional collagen deposition. This nuanced understanding has the capacity to optimize HT diagnosis, enabling clinicians to discern intricate morphological changes that might serve as key diagnostic markers. Furthermore, our observation of immunological synapses involving lymphocytes, plasmacytes, and thyrocytes within the stromal context offers a compelling avenue for novel, effective predictive, diagnostic, and treatment-monitoring approaches. This intricate immune interplay may provide clinicians with dynamic biomarkers that reflect the disease’s activity and guide treatment decisions with greater precision. Lastly, the notable presence of plasma cells exhibiting enhanced capability for antibody production could also lead to the development of tailored interventions that modulate antibody responses, offering a fresh direction for treatment approaches that address underlying immune dysregulation.

The thyroid glands of HT patients are characterized by increased blood flow and vascularization [27]. Angiogenesis and related angiogenic factors, such as vascular endothelial growth factor (VEGF), play a pivotal role in thyroid cell growth and function, even in thyroid pathology [28,29,30,31,32,33,34]. VEGF is one of the main factors responsible for the increased vascularization of the gland. Several studies have demonstrated elevated serum VEGF levels in patients with HT [27,32,35]. Interestingly, a recent study has reported a connection between VEGF expression levels and the severity of HT. Patients with severe HT seemed to highly express VEGF, enhancing angiogenesis [35]. In the same context, previous research revealed a significant increase in serum VEGF levels and intrathyroidal vascular area in patients with untreated goitrous HT compared to healthy subjects, indicating active intrathyroidal angiogenesis in HT [32]. Anti-VEGF agents are currently striving to establish a prominent presence in the landscape of tumor treatment, including head and neck squamous cell carcinoma (HNSCC) [36,37] and potentially papillary thyroid carcinoma (PTC) [38]; therefore, their significance could extend to HT regimens.

As it is known, VEGF is secreted by thyrocytes in response to TSH [39,40,41,42]. Conceivably, it is expected that serum TSH concentrations in HT, which are decreased remarkably after levothyroxine treatment, especially when euthyroid state has been achieved [32,43], might be positively correlated with serum VEGF levels [27,32]. Considering that most HT cases evolve into hypothyroidism with elevated serum TSH levels [44], the increased production of VEGF by thyrocytes in response to TSH stimuli may explain the abundance of smaller vessels. However, low levels of VEGF in HT patients compared to healthy controls have also been reported in another study where patients were euthyroid due to replacement therapy [43]. It remains to be clarified whether the administration of thyroid hormone replacement therapy in HT impacts the vascular ultrastructure [32].

Patients with subclinical hypothyroidism caused by HT are characterized by generalized endothelial dysfunction, leading to an increased prevalence of atherosclerotic lesions and cardiovascular events [45]. These conditions are partly associated with chronic inflammation and impaired nitric oxide availability, resulting in increased oxidative stress [46]. Overall, there seems to be a connection between endothelial dysfunction and high levels of TSH in subclinical hypothyroidism caused by HT [46]. Interestingly, HT is often comorbid with several forms of autoimmune vasculitides, e.g., antineutrophil cytoplasmic antibody (ANCA)-associated vasculitis (AAV), ANCA-associated small-vessel vasculitis (ANCA SVV), and systemic vasculitis (SV) [47,48,49]. Moreover, HT is suggested to be linked with a marker of atherosclerosis, namely the pulse wave velocity, a correlation that leads to arterial stiffness with structural changes such as thinning and fragmentation of elastin, medial smooth muscle cell necrosis, and fibrosis [50]. Nevertheless, the described alterations are generalized and have yet to be ascribed to the thyroid. Similarly, endothelium dysfunction in HT patients has also been correlated with TPO antibodies [51].

Angiogenesis is greatly supported by fibroblasts, which are reported to be a key element in the process [52]. Fibrosis is present to different extents in HT, with numerous fibroblasts and large amounts of collagen fibers [53]. Fibroblasts could promote extracellular matrix remodeling, facilitate tissue supply with growth factors, and, finally, differentiate into pericytes when new vessels are formed. Fibroblasts are also known to enhance the stability of nascent capillaries [52]. Our research hints at a fibroblastic activity directly linked to vessel homeostasis; thus, this connection might have an important role in the pathogenesis of HT. Parallel endeavors are being made in our lab toward this trajectory. Lastly, the present case-series ultrastructural study has two main limitations: the small sample size and the lack of acquisition and correlation between VEGF and TSH measurements in serum before replacement therapy.

## 5. Conclusions

This preliminary study presents a noteworthy advancement in our understanding of HT by providing updated insights into its ultrastructural elements. Particularly significant among the main TEM findings was the identification of small vessels exhibiting minor damage and larger vessels displaying more extensive impairments. Furthermore, the observation of atypia in thyrocytes adds an intriguing layer of complexity. The elucidation of these ultrastructural intricacies stands as a pivotal step toward unraveling the underlying mechanisms driving HT, ultimately paving the way for more targeted and effective therapeutic interventions.

## Data Availability

Data are available from authors upon reasonable request.
